# Higher dietary butyrate intake is associated with better cognitive function in older adults: evidence from a cross-sectional study

**DOI:** 10.3389/fnagi.2025.1522498

**Published:** 2025-03-28

**Authors:** Jiayu Tu, Jun Zhang, Gang Chen

**Affiliations:** Department of Anesthesiology, Sir Run Run Shaw Hospital, Zhejiang University, Hangzhou, China

**Keywords:** cognitive function, butyrate intake, short-chain fat acids, NHANES, cross-sectional study

## Abstract

**Background:**

Studies indicate that butyrate can enhance memory and cognitive functions in mice by inhibiting neuroinflammation and neuronal apoptosis. Elevated fecal butyrate levels in older individuals with mild cognitive impairment correlate with reduced levels of Aβ-42, an Alzheimer’s disease biomarker. This study investigated the relationship between butyrate consumption and cognitive performance in older adults, which remains to be elucidated.

**Methods:**

This study employed a cross-sectional, observational design to analyze data gathered from 2,078 participants enrolled in the 2011–2014 US National Health and Nutrition Examination Survey (NHANES). Butyrate intake was determined based on two 24-h dietary assessments. To evaluate cognitive function, three tests were administered: the Animal Fluency Test (AFT) to assess executive function, the Digit Symbol Substitution Test (DSST) for measuring processing speed, and the Consortium to Establish a Registry for Alzheimer’s disease (CERAD) subtest for assessing memory. Z scores were computed for each test and overall cognitive performance. Multivariate linear regression models and a generalized additive model (GAM) were used to examine the correlation between butyrate consumption and mental functions. Finally, subgroup analyses and interaction tests were used to verify the robustness of the associations.

**Results:**

The NHANES study encompasses two surveys conducted between 2011 and 2014 that involved 2,078 participants aged 60 years or older. Higher dietary butyrate consumption had a positive correlation between superior performance on DSST, AFT, CERAD-Immediate Recall Test, and Z scores. The participants in the upper quartile of butyrate intake had significantly higher DSST (*β* = 1.60, 95% CI: 0.04–3.17), AFT scores (*β* = 0.99, 95% CI: 0.37–1.60), and Z scores (*β* = 0.09, 95% CI: 0.01–0.17) than individuals in the lowest quartile even after adjusting for potential confounders. Finally, no notable interactions were observed within the groupings. Finally, in subgroup analyses, BMI was found to influence the positive association between butyrate and DSST with Z score, and hypertension also influenced the association between butyrate and DSST.

**Conclusion:**

Higher butyrate intake in individuals aged ≥60 years was linked to better cognitive functioning. This could potentially contribute to maintaining brain function during aging.

## Introduction

The growing trend of population aging is resulting in a surge in the prevalence of cognitive decline associated with aging (Brito et al., 2023). According to 2023 reports, 6.7 million individuals aged 65 and older in the United States have Alzheimer’s disease (AD), with projections indicating a rise to 13.8 million by 2060 (Alzheimers Dement, 2023). Cognitive impairment is increasingly recognized as a major public health challenge worldwide, and can significantly affect an individual’s daily life and independence. For example, studies have shown that individuals with subjective cognitive decline may experience significant quality of life decline and functional limitations within 6 months (Hendriksen et al., 2024). The higher prevalence of cognitive impairment among older adults may be related to a variety of factors, including chronic disease, psychosocial factors, and lifestyle. For example, studies have found that up to 76.0% of older adults living in foster care in the French West Indies have severe cognitive impairment (Boucaud-Maitre et al., 2024). In addition, cognitive decline may also be associated with specific biomarkers, such as amyloid PET scan disclosure in individuals with subjective cognitive decline can reveal the risk of future cognitive decline (Hendriksen et al., 2024). In contrast, in patients with cerebral small vessel disease, the mismatch between high white matter signal and cognitive function also suggests that cognitive decline may be associated with cerebrovascular lesions (Zeng et al., 2024). These findings emphasize the multifactorial nature and complexity of cognitive decline and the importance of early identification and intervention. However, to date, knowledge of cognitive impairment remains limited.

The role of the gut microbiome in neurodegenerative diseases is increasingly being acknowledged (Loh et al., 2024; Alpino et al., 2024). The fermentation of fiber-rich diets results in the production of short-chain fatty acids (SCFAs), primarily consisting of saturated fatty acids with 1 to 6 carbon atoms. The dosage of SCFAs, particularly butyrate, may be critical in determining their effects on psychophysiological and behavioral processes (Dalile et al., 2019). Recent studies indicate that butyrate can enhance memory and cognitive functions in mice by inhibiting microglia-mediated neuroinflammation and neuronal apoptosis (Wang et al., 2023; Wei et al., 2023). In a vascular dementia model, administration of butyrate-producing bacteria resulted in increased butyrate levels in the feces and the brain, significantly reduced cognitive deficits, and histopathological changes in the hippocampus (Liu et al., 2015). Butyrate may also exert neuroprotective effects by increasing neurotrophic factor levels and improving mitochondrial autophagy dysregulation (Guo et al., 2025). As the global population ages, cognitive decline has become an important public health issue. In recent years, more and more studies have shown that the gut microbiota and its metabolites (e.g., butyrate) play an important role in the aging process. Transplantation of gut microbes from older mice to younger mice resulted in inflammation of the gut and brain with cognitive decline, and further studies found that older gut microbes produced fewer butyrate-producing bacteria (Mishra et al., 2024). In addition, a study of older adults with mild cognitive impairment found that elevated levels of butyrate in participants’ feces were associated with lower levels of the AD biomarker Aβ-42 (Nagpal et al., 2019). Therefore, studying the relationship between butyrate intake and cognitive function in older adults is important for understanding the mechanisms of cognitive changes during aging.

The present study investigated the relationship between butyrate and cognitive function in older adults by analyzing data from NHANES and a representative group of older adults to address gaps in previous studies and reveal the potential benefits of dietary modifications in preventing cognitive decline.

## Methods

### Data sources and study population

The National Center for Health Statistics conducts the National Health and Nutrition Examination Survey (NHANES), which gathers vital information about the nutritional status and overall health of citizens in the United States of America. This survey collects data on various aspects, including population characteristics, dietary supplements, laboratory tests, physical examinations, and questionnaire responses (Elnakib et al., 2022; Saint-Maurice et al., 2022; Kim et al., 2023). The NHANES ensures that the sample accurately represents the US civilian population by employing a stratified, multi-stage sampling method. All participants in the study provided written consent, and the Research Ethics Committee of the National Health and Demographic Survey granted its official endorsement. For further details about NHANES, you can visit http://www.cdc.gov/nchs/nhanes.htm.

**Figure 1:**
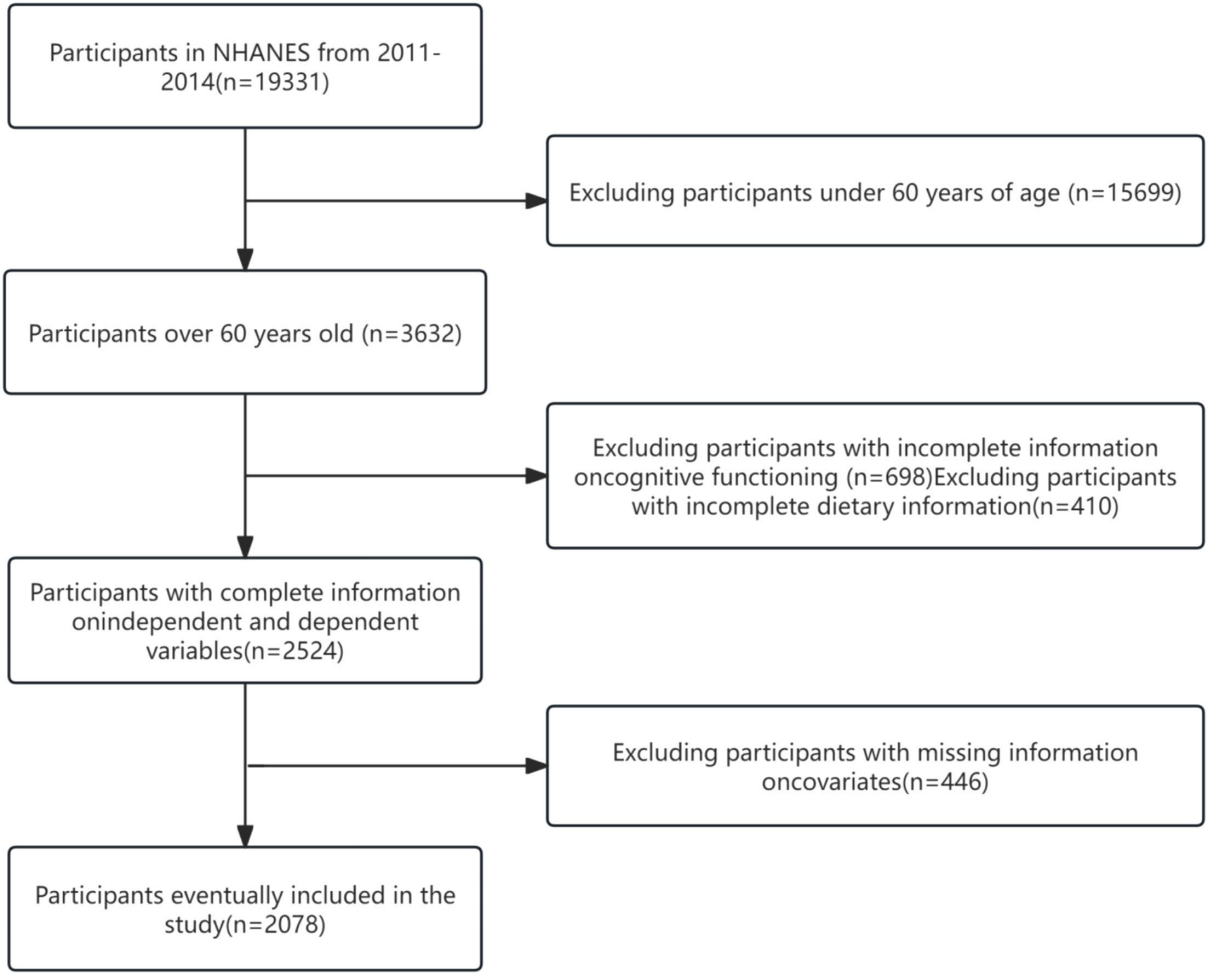
Flowchart of patient screening in retrospective analysis.

Our study collected data from NHANES cycles conducted from 2011 to 2014 (*n* = 19,931). To ensure the validity of the findings, we selected older adults who were older than 60 years of age (*n* = 16,299). Participants missing complete cognitive information (*n* = 698) and missing information on butyrate intake (*n* = 410) were excluded from the study analyses. In addition, we excluded participants missing information on covariates (*n* = 446). Ultimately, we analyzed data from 2,078 participants aged 60 years or older (Ghaferi et al., 2021). Detailed inclusion and exclusion labeling can be found in Figure 1.

### Dietary butyrate intake

The NHANES employed a comprehensive 24-h dietary recall questionnaire to document individual food consumption throughout the previous day. Each participant completed two 24-h dietary recall interviews, and the data obtained from these interviews were used to assess their daily butyrate intake. The first interview occurred at a portable testing facility, and a further interview may be arranged via telephone within a timeframe of 3 to 10 days. Dietary butyrate intake was estimated from participants’ reported food intake. We used the U.S. Department of Agriculture (USDA) food composition database in conjunction with data on the butyrate content of foods to calculate daily butyrate intake from participant-reported food intake. This calculation is done after data collection. The results were obtained by calculating the average butyrate consumption from both interviews for each individual and subsequently classifying them according to this value.

### Cognitive function

The NHANES study incorporated cognitive assessments aimed at evaluating memory and executive functioning. The researchers assessed the ability of the study participants to learn new words using the Consortium to Establish a Registry for Alzheimer’s Disease-Immediate Recall Test (CERAD-IRT) and CERAD-Delayed Recall Test (DRT) tests, which were created by the Alzheimer’s Disease Word Study Registry Consortium. The participants were presented with a roster of 10 disparate things and instructed to recall as many terms as possible. Their scores from the initial three attempts were subsequently combined. An 8–10-min period elapsed before administering a delayed recall test. The participants of the Animal Fluency Test (AFT) were instructed to generate as many animal names as possible within a 1-min time frame. Each correctly named animal was awarded one score. This test aimed to assess participants’ linguistic and executive skills. The participants’ processing speed and executive functioning were evaluated through the use of the Digit Symbol Substitution Test (DSST). This test required the participants to rapidly associate symbols with their respective digits, utilizing a given legend. The final stage for computing Z scores was taking the mean of four standardized measures: the DSST, the AFT, the CERAD-IRT, and the CERAD-DRT (Jia et al., 2024).

### Covariates

Our study included individuals from several ethnic backgrounds, including non-Hispanic black, white, Mexican American, and other races. Participants’ marital statuses were divided into two groups: married, and single. There were three tiers of educational achievement: fewer than 9 years of formal education, 9 to 12 years of formal education, and 13 years of formal education or beyond. The poverty-to-income ratio (PIR) was split into three categories: low (<1.3), moderate (1.3–3.5), and high (>3.5). The participants were categorized into three groups: current smokers, former smokers (those who had quit smoking after consuming 100 cigarettes), and never smokers (those who had smoked less than 100 cigarettes in total). Similarly, individual patterns of alcohol use were categorized into three distinct groups: abstainers (those who had consumed fewer than 12 alcoholic beverages in their lifetime), current drinkers, and former drinkers.

### Statistical analysis

Chi-square and one-way ANOVA tests were used to compare participant baseline characteristics. For continuous data, we employed the median and interquartile range or standard deviation as measures of central tendency and dispersion, respectively. For categorical variables, we utilized total proportions and percentages as representations. The issue of multiple comparisons was resolved by implementing the Bonferroni adjustment.

Butyrate consumption was divided into four groups, with the lowest category being used as the reference. We conducted a mixed-category multivariate linear regression analysis to examine the correlation between butyrate intake and cognitive function. Model 1 served as our basic model, lacking any adjustments. In contrast, Model 2 incorporated adjustments for age, sex, PIR, race/ethnicity, and educational level. Extending the adjustments of Model 2, Model 3 further accounted for body mass index (BMI), marital status, smoking habits, alcohol consumption, diabetes mellitus, hypertension, cardiovascular disease, stroke, cholesterol levels, creatinine, and alanine aminotransferase (ALT). The study examined the relationship between the intake of butyrate and cognitive test performance using the cubic spline method. The linear trend analysis was used to investigate the categorical components. We performed subgroup analyses by employing binary linear regression models. These models were stratified and classified based on clinical thresholds or quartiles. After that, we conducted interaction tests and carried out effect-adjusted tests for subgroup measures alongside likelihood ratio tests.

All statistic computations were executed using R software (version 4.1.1, R Foundation, Vienna, Austria). A two-sided *p*-value <0.05 was established as the criterion for statistical significance. To ensure data integrity, we excluded all participants with missing data.

## Results

### Baseline participant characteristics

Table 1 outlines the baseline traits of the study participants according to their dietary butyrate consumption. Based on a quartile analysis of their butyrate intake, the participants were categorized into four groups: Q1 (consuming ≤0.341 g/day), Q2 (0.341 < consuming ≤0.68 g/day), Q3 (0.68 < consuming ≤1.172 g/day), and Q4 (consuming >1.172 g/day). The average age of the participants was 69.39 ± 6.73 years, 1,093 (52.60%) were women, and 1,070 (51.49%) identified as non-Hispanic white. Those with higher butyrate intake tended to be male, with higher education and economic income and lower prevalence of hypertension and diabetes (*p* < 0.05). Cognitive scores were significantly higher in the group with higher butyrate intake than in the group with lower intake, as assessed by DSST, AFT, CERAD-IRT, and Z scores (*p* < 0.01). Regarding the biochemical indicators, there were no noticeable differences across the groups.

**Table 1:** Baseline characteristics of the study population from 2011–2014.

	Total	Butyrate intake quartiles (gm/d)				*p*-value
		Q1	Q2	Q3	Q4	
N	2078	518	516	524	520	
Age, years	69.39 ± 6.73	68.89 ± 6.55	68.78 ± 6.58	69.87 ± 6.88	70.01 ± 6.81	0.003
Gender, *n* (%)						0.057
Male	985 (47.40)	233 (44.98)	229 (44.38)	253 (48.28)	270 (51.92)	
Female	1,093 (52.60)	285 (55.02)	287 (55.62)	271 (51.72)	250 (48.08)	
BMI, kg/m^2^						0.201
Normal	537(25.84)	151 (29.15)	122 (23.64)	129 (24.62)	135 (25.96)	
Overweight	731 (35.18)	181 (34.94)	189 (36.63)	171 (32.63)	190 (36.54)	
Obese	810 (38.98)	186 (35.91)	205 (39.73)	224 (42.75)	195 (37.50)	
Race, *n* (%)						<0.001
Non-Hispanic white	1,070 (51.49)	184 (35.52)	231 (44.77)	308 (58.78)	347 (66.73)	
Non-Hispanic black	478 (23.00)	167 (32.24)	133 (25.78)	99 (18.89)	79 (15.19)	
Mexican American	167 (8.04)	42 (8.11)	51 (9.88)	40 (7.63)	34 (6.54)	
Others	363 (17.47)	125 (24.13)	101 (19.57)	77 (14.69)	60 (11.54)	
Education level, *n* (%)						<0.001
Less than high school	196 (9.43)	57 (11.00)	60 (11.63)	50 (9.54)	29 (5.58)	
High school	275 (13.23)	98 (18.92)	71 (13.76)	64 (12.21)	42 (8.08)	
More than high school	1,607 (77.33)	363 (70.08)	385 (74.61)	410 (78.24)	449 (86.35)	
Marital status, *n* (%)						0.389
Married	1,222 (58.81)	292 (56.37)	299 (57.95)	322 (61.45)	309 (59.42)	
Separated	856 (41.19)	226 (43.63)	217 (42.05)	202 (38.55)	211 (40.58)	
PIR, *n* (%)						<0.001
< 1.3	577 (27.77)	178 (34.36)	169 (32.75)	117 (22.33)	113 (21.73)	
1.3–3.5	823 (39.61)	204 (39.38)	193 (37.40)	218 (41.60)	208 (40.00)	
>3.5	678 (32.63)	136 (26.25)	154 (29.84)	189 (36.07)	199 (38.27)	
Smoking status, n (%)						0.424
Never	246 (11.84)	72 (13.90)	58 (11.24)	54 (10.31)	62 (11.92)	
Former	804 (38.69)	187 (36.10)	195 (37.79)	208 (39.69)	214 (41.15)	
Current	1,028 (49.47)	259 (50.00)	263 (50.97)	262 (50.00)	244 (46.92)	
Alcohol status, *n* (%)						0.002
Never	1,443 (69.44)	326 (62.93)	358 (69.38)	367 (70.04)	392 (75.38)	
Former	328 (15.78)	99 (19.11)	77 (14.92)	80 (15.27)	72 (13.85)	
Current	307 (14.77)	93 (17.95)	81 (15.70)	77 (14.69)	56 (10.77)	
Hypertension, *n* (%)						0.006
Yes	1,309 (62.99)	341 (65.83)	340 (65.89)	333 (63.55)	295 (56.73)	
No	769 (37.01)	177 (34.17)	176 (34.11)	191 (36.45)	225 (43.27)	
Diabetes, *n* (%)						<0.001
Yes	489 (23.53)	152 (29.34)	130 (25.19)	106 (20.23)	101 (19.42)	
No	1,589 (76.47)	366 (70.66)	386 (74.81)	418 (79.77)	419 (80.58)	
Cardiovascular disease, *n* (%)						0.618
Yes	376 (18.09)	99 (19.11)	97 (18.80)	85 (16.22)	95 (18.27)	
No	1702 (81.91)	419 (80.89)	419 (81.20)	439 (83.78)	425 (81.73)	
Cognitive score						
DSST	47.36 ± 16.77	44.18 ± 16.43	46.28 ± 17.37	48.31 ± 16.74	50.66 ± 15.85	<0.001
AFT	16.96 ± 5.44	15.84 ± 5.12	16.47 ± 5.35	17.40 ± 5.74	18.14 ± 5.25	<0.001
CERAD-IRT	19.18 ± 4.54	18.93 ± 4.47	18.99 ± 4.63	19.13 ± 4.66	19.64 ± 4.37	0.049
CERAD-DRT	6.06 ± 2.28	5.99 ± 2.20	6.07 ± 2.33	6.03 ± 2.27	6.14 ± 2.34	0.762
Z score	−0.01 ± 0.78	−0.13 ± 0.73	−0.05 ± 0.80	0.02 ± 0.80	0.13 ± 0.76	<0.001
Cholesterol, mmol/L	4.95 ± 1.12	4.93 ± 1.16	5.04 ± 1.14	4.93 ± 1.06	4.92 ± 1.11	0.274
Creatinine, umol/L	89.72 ± 51.87	89.87 ± 41.11	89.67 ± 44.49	87.11 ± 38.30	92.27 ± 75.03	0.459
ALT, U/L	22.21 ± 13.01	22.71 ± 16.44	22.18 ± 11.38	22.05 ± 9.57	21.89 ± 13.66	0.758
AST, U/L	25.16 ± 11.07	25.58 ± 13.85	24.92 ± 10.15	25.04 ± 7.90	25.10 ± 11.55	0.790

All results were analyzed using weighted. Continuous variables are expressed as mean (SD) or median (interquartile range) and categorical variables are expressed as frequency (percentage). BMI, body mass index; PIR, poverty income ratio; ALT, alanine amiotransferase; AST, aspartate aminotransferase; DSST, digit symbol substation test; AFT, animal fluency test; CERAD, consortium to establish a registry for Alzheimer’s disease; CERAD-IRT, immediate recall in CERAD trial; CERAD-DRT, delayed recall in CERAD trial.

### Relationship between butyrate intake and cognitive function

Table 2 presents the multivariate linear regression analysis of the association between butyrate intake and cognitive function in elderly participants. Higher butyrate intake was positively associated with cognitive scores. In the crude model, participants in the highest Q4 group exhibited higher cognitive scores compared to those in the lowest Q1 group (DSST: *β* = 6.48, 95% CI: 4.46–8.50; AFT: *β* = 2.31, 95% CI: 1.65–2.96; and Z scores: *β* = 0.26, 95% CI: 0.16–0.35). In addition, cognitive scores increased progressively with increasing butyrate intake (P for trend <0.001). In Model 3, after adjusting for various covariates such as age, sex, race, PIR, marital status, education level, BMI, smoking, drinking, hypertension, diabetes, cardiovascular disease, ALT, AST, creatinine, and cholesterol, participants in the Q4 group had higher DSST (*β* = 1.60, 95% CI: 0.04–3.17), AFT scores (*β* = 0.99, 95% CI: 0.37–1.60), and Z scores (*β* = 0.09, 95% CI: 0.01–0.17), compared with those in Q1. In addition, cognitive scores including AFT and Z scores increased progressively with increasing butyrate intake (P for trend <0.05).

**Table 2:** Multivariable linear regression to assess the association of Butyrate intake with cognitive function.

	DSST		AFT		CERAD-IRT		CERAD-DRT		Z score	
	β (95% CI)	*P*-value	β (95% CI)	*P*-value	β (95% CI)	*P*-value	β (95% CI)	*P*-value	β (95% CI)	*P*-value
Model 1: without adjustment										
Butyrate (mg/day)	4.15 (2.86, 5.44)	<0.001	1.59 (1.18, 2.01)	<0.001	0.46 (0.11, 0.81)	0.011	0.09 (−0.09, 0.27)	0.332	0.17 (0.11, 0.23)	<0.001
Butyrate quartile										
Q1	0.00 (Reference)		0.00 (Reference)		0.00 (Reference)		0.00 (Reference)		0.00 (Reference)	
Q2	2.10 (0.08, 4.12)	0.042	0.64 (−0.02, 1.29)	0.057	0.06 (−0.49, 0.61)	0.832	0.08 (−0.20, 0.36)	0.586	0.07 (−0.02, 0.17)	0.132
Q3	4.13 (2.12, 6.15)	<0.001	1.56 (0.91, 2.21)	<0.001	0.20 (−0.35, 0.75)	0.474	0.03 (−0.25, 0.31)	0.818	0.15 (0.05, 0.24)	0.002
Q4	6.48 (4.46, 8.50)	<0.001	2.31 (1.65, 2.96)	<0.001	0.71 (0.16, 1.26)	0.012	0.14 (−0.13, 0.42)	0.309	0.26 (0.16, 0.35)	<0.001
P for trend	2.15 (1.51, 2.79)	<0.001	0.78 (0.58, 0.99)	<0.001	0.23 (0.05, 0.40)	0.011	0.04 (−0.05, 0.13)	0.388	0.08 (0.06, 0.11)	<0.001
Model 2: adjusted for age, gender, race, PIR, and education level										
Butyrate (mg/day)	1.26 (0.24, 2.27)	0.015	0.79 (0.40, 1.18)	<0.001	0.27 (−0.07, 0.60)	0.117	0.02 (−0.15, 0.19)	0.784	0.07 (0.02, 0.12)	0.005
Butyrate quartile										
Q1	0.00 (Reference)		0.00 (Reference)		0.00 (Reference)		0.00 (Reference)		0.00 (Reference)	
Q2	0.52 (−1.02, 2.05)	0.511	0.18 (−0.42, 0.77)	0.565	−0.11 (−0.62, 0.39)	0.66	0.00 (−0.26, 0.26)	0.99	0.01 (−0.07, 0.09)	0.806
Q3	1.27 (−0.29, 2.82)	0.111	0.74 (0.14, 1.35)	0.016	0.03 (−0.48, 0.54)	0.909	−0.02 (−0.28, 0.24)	0.877	0.05 (−0.03, 0.13)	0.191
Q4	2.07 (0.49, 3.65)	0.011	1.09 (0.48, 1.71)	<0.001	0.41 (−0.11, 0.93)	0.124	0.04 (−0.22, 0.31)	0.747	0.11 (0.03, 0.19)	0.008
P for trend	0.70 (0.19, 1.20)	0.007	0.39 (0.19, 0.58)	<0.001	0.13 (−0.03, 0.30)	0.1121	0.01 (−0.07, 0.09)	0.8172	0.04 (0.01, 0.06)	0.0046
Model 3: adjusted as for Model 2, additionally adjusted for marital status, BMI, smoking, alcohol, hypertension, diabetes, cardiovascular disease, ALT, AST, creatinine, and cholesterol										
Butyrate (mg/day)	1.02 (0.02, 2.02)	0.045	0.73 (0.34, 1.12)	<0.001	0.23 (−0.10, 0.57)	0.173	0.00 (−0.17, 0.17)	0.981	0.06 (0.01, 0.11)	0.017
Butyrate quartile										
Q1	0.00 (Reference)		0.00 (Reference)		0.00 (Reference)		0.00 (Reference)		0.00 (Reference)	
Q2	0.20 (−1.31, 1.72)	0.791	0.12 (−0.47, 0.72)	0.690	−0.13 (−0.64, 0.37)	0.606	−0.02 (−0.27, 0.24)	0.908	−0.00 (−0.08, 0.08)	0.992
Q3	0.75 (−0.78, 2.29)	0.336	0.65 (0.04, 1.25)	0.036	−0.02 (−0.54, 0.49)	0.931	−0.07 (−0.33, 0.20)	0.620	0.03 (−0.05, 0.11)	0.415
Q4	1.60 (0.04, 3.17)	0.044	0.99 (0.37, 1.60)	0.002	0.35 (−0.17, 0.88)	0.186	0.01 (−0.26, 0.27)	0.968	0.09 (0.01, 0.17)	0.028
P for trend	0.54 (0.04, 1.03)	0.034	0.35 (0.15, 0.54)	<0.001	0.12 (−0.05, 0.28)	0.167	−0.00 (−0.09, 0.08)	0.940	0.03 (0.00, 0.06)	0.020

CI, confidence interval; BMI, body mass index; PIR, poverty income ratio; ALT, alanine amiotransferase; AST, aspartate aminotransferase; DSST, digit symbol substation test; AFT, animal fluency test; CERAD, consortium to establish a registry for Alzheimer’s disease; CERAD-IRT, immediate recall in CERAD trial; CERAD-DRT, delayed recall in CERAD trial.

### Dose–response relationships

**Figure 2:**
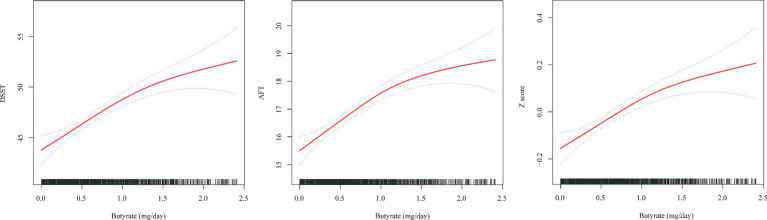
Association between butyrate (g/day) intake and cognitive impairment in an elderly population. The solid red line represents the smooth curve fit between variables. Blue bands represent the 95% confidence interval from the fit. All analyses were adjusted for age, gender, race, PIR, education level, marital status, BMI, smoking, alcohol, hypertension, diabetes, cardiovascular disease, stroke history, ALT, AST, creatinine, and cholesterol.

To further explore the dose–response association between dietary butyrate intake and cognitive impairment in the elderly population, GAM analysis was used to visualize the association between butyrate intake and DSST, AFT, and Z scores (Figure 2). Following comprehensive adjustments, the results revealed a distinct association between the consumption of butyrate and cognitive function scores, showcasing a link that varies based on the dosage.

### Subgroup analysis

Our analyses showed that as far as the relationship between butyrate and AFT was concerned, it was positively associated across subgroups (age, gender, BMI, race, hypertension, diabetes, and cardiovascular disease) and the interactions showed that there was no significant interaction between the various factors and butyrate. (P for all interactions ≥0.05, Table 3). Furthermore, in Supplementary Tables 1, 2, positive associations between butyrate and DSST and Z score were similarly found. The interaction suggests that BMI and hypertension mediated the association between butyrate and DSST. Similarly, BMI mediated a positive association between butyrate and Z score.

**Table 3:** Subgroup analysis of the association between dietary butyrate intake and AFT.

	β (95% CI)	*P*	P for interaction
Age			0.241
<70	1.092(0.563, 1.621)	<0.001	
≥70	0.837(0.238, 1.436)	0.006	
Gender			0.868
Male	0.903(0.348, 1.458)	0.001	
Female	1.007(0.427, 1.587)	<0.001	
BMI			0.713
Obese	1.187(0.550, 1.824)	<0.001	
Normal	1.334(0.503, 2.165)	0.002	
Overweight	0.338(−0.336, 1.012)	0.325	
Underweight	3.229(−5.862,12.319)	0.429	
Race			0.407
Non-Hispanic white	0.285(−0.250, 0.820)	0.296	
Non-Hispanic black	1.859(0.959, 2.760)	<0.001	
Mexican American	0.765(−0.793, 2.324)	0.333	
Others	1.021(0.063, 1.979)	0.037	
Hypertension			0.137
Yes	1.218(0.708, 1.727)	<0.001	
No	0.555(−0.088, 1.198)	0.090	
Diabetes			0.290
No	0.816(0.357, 1.276)	<0.001	
Yes	1.41(0.620, 2.201)	<0.001	
Cardiovascular disease			0.429
No	1.019(0.574, 1.464)	<0.001	
Yes	0.544(−0.353, 1.441)	0.234	

## Discussion

This study analyzed data from a cross-sectional survey conducted between 2011 and 2014, focusing on older individuals. The findings revealed a substantial connection between butyrate intake and enhanced cognitive scores, even after accounting for potential confounding factors. Additionally, a notable dose-dependent relationship existed between butyrate intake and cognitive function in the Q1-Q4 intervals. The relationship between butyrate intake and AFT exhibited a significant nonlinear pattern, with an inflection point at approximately 1.73 g/day of butyrate intake. Before this point, AFT increased gradually with increasing butyrate intake; however, beyond the inflection point, the curve tended to decrease, suggesting that there might be an optimal range of butyrate intake for cognitive function beyond which additional intake may negatively impact cognitive function. For patients aged <80 years with hypertension without diabetes or stroke, butyrate had significant cognitive protective factors, although no significant differences were observed in the overall study population.

The relationship between AD and gut microbes has been extensively investigated in clinical studies, demonstrating a significant correlation between cognitive function and the prevalence of butyrate-producing bacteria (Verhaar et al., 2021; Hung et al., 2022). A clinical study involving 56 observers revealed a significant correlation between increased free water imaging in gray matter regions and a scarcity of butyrate-producing microorganisms in individuals belonging to the groups with AD and mild cognitive impairment (Yamashiro et al., 2024). Individuals with obesity typically exhibit imbalances in their gut microbiota, characterized by an increase in Aspergillus and a decrease in Clostridium butyrate levels. These imbalances have been strongly linked to cognitive decline. Furthermore, research indicates that oral Clostridium butyrate supplementation can effectively improve cognitive function in these individuals (Zheng et al., 2024). A prospective cohort study involving 135 patients, including those with cognitive normality, subjective cognitive decline, or mild cognitive impairment (MCI), found that patients with prodromal AD phenotype had lower levels of SCFA-producing bacteria, particularly butyrate-producing ones, compared to the control group with cognitive normality, with similar results observed in those with prior MCI (Liu et al., 2022; Liu et al., 2019). Research has demonstrated that vitamins B, D, and E possess neuroprotective qualities in the context of AD and Parkinson’s disease (Jia et al., 2024; Dysken et al., 2014; Wang et al., 2021; Samson et al., 2022; Zhou, 2023). Moreover, research on polyunsaturated fatty acids (PUFAs) has shown that increased levels of PUFAs and continuous supplementation with PUFAs over a period of time might potentially improve motor dysfunction and decelerate the advancement of cognitive decline in patients with AD (Chiu et al., 2008; Avallone et al., 2019; Chu et al., 2022). Clinical studies have shown that omega-3 PUFA supplements can enhance the presence of bacteria that are responsible for the production of butyrate and Mycobacterium anisopliae (Costantini et al., 2017; Zhuang et al., 2018; Zheng et al., 2021).

Clostridium butyrate (Cb), a member of the Clostridium genus and classified as a bacillus, is notable for its capability to produce butyrate, which has been associated with potential benefits for neurological ailments and the aging process (Stoeva et al., 2021). Research indicates that administering Cb to AD mice for 4 weeks effectively prevents A*β* accumulation, reduces microglia activation, and mitigates cognitive impairments (Sun et al., 2020). Oral administration of Cb improved motor functions impaired by MPTP, reduced dopaminergic neuron loss, mitigated synaptic dysfunction, and inhibited microglia activation in mice (Sun et al., 2021). Additionally, it significantly alleviated cognitive impairments and neuronal damage in a mouse model of sepsis-associated encephalopathy (Liu et al., 2020). Systematic transplantation and grafting of fecal microbiota from healthy wild-type mice to animals with AD improved the development of amyloid β-plaques and neurofibrillary tangles, decreased neuroglial reactions, and relieved cognitive impairment (Kim et al., 2020). Administering butyrate directly enhances cognitive function in mice with AD (Wang et al., 2022; Zhou et al., 2023). A comprehensive investigation incorporating both preclinical and clinical research discovered that a maternal diet low in fiber negatively affected cognitive function and synaptic plasticity in the offspring. Furthermore, it was determined that butyrate consumption, but not propionate, could reverse this impairment. Subsequent mechanistic studies revealed that histone deacetylase 4 is the primary mediator of butyrate-dependent neurocognitive improvement (Yu et al., 2020).

Although the exact mechanisms linking butyrate intake to cognitive increase remain unclear, our results are biologically plausible. Prior research has demonstrated a negative relationship between serum quinolinic acid levels and cognitive function and cortical gray matter reduction in human obesity. Quinolinic acid is known to harm neurons, alter intracellular signaling in dendritic spines, and decrease brain-derived neurotrophic factor (BDNF). However, butyrate, a gut microbiota metabolite, prevents quinolinic acid-induced damage by epigenetically enhancing H3K18ac on BDNF promoters, offsetting low BDNF levels and improving cognition (Ge et al., 2023). The oral administration of butyrate in mice exposed to lead improves cognitive memory by inhibiting STAT3 signaling in microglia and stimulating ACSS2 expression in hippocampal neurons. This increases acetyl-CoA levels, restoring H3K9ac deposition and BDNF expression (Li et al., 2024). Reduced butyrate production by the aged gut microbiota leads to decreased signaling of FFAR2/3, inhibiting mucin formation and increasing permeability, inflammation, and brain abnormalities (Mishra et al., 2024).

In our supplemental analyses, butyrate intake was found to be positively associated with Z-scores in participants younger than 70 years of age, whereas this relationship was no longer significant in participants older than 70 years of age. The possible reasons behind this are as follows: the composition and function of the intestinal microbiota undergoes significant changes with age, which may lead to less efficient production and absorption of butyrate (Ling et al., 2022). In addition, in older adults 70 years of age and older, chronic inflammation, oxidative stress, or other neurodegenerative pathologic changes that may be present may mask the potential benefits of butyrate (Khansari et al., 2009; Mou et al., 2022).

Employing a multifaceted approach, this study comprehensively assessed cognitive function through various tests, including CERAD-DRT, CERAD-IRT, DSST, AFT, and Z scores. To ensure a rigorous analysis, we accounted for confounders identified in prior research. To further investigate the possible relationship between butyrate consumption and cognitive performance, a dose–response study was also conducted.

Nonetheless, it is crucial to recognize the study’s limitations. First, given its cross-sectional design, determining the precise temporal relationship between butyrate intake and cognitive function remains elusive. Randomized controlled trials are essential for further validating our findings. Second, although efforts were made to account for potential confounding factors, it was not possible to completely remove the chance of reverse causation or residual confounding. Third, our reliance on self-reported 24-h dietary recalls for butyrate intake data introduced the risk of measurement and recall inaccuracies. Fourth, due to the limitations of the retrospective study, we did not collect information on whether participants additionally ingested butyrate supplements; we should take this factor into account in future studies and should be mindful of the scope of application in terms of interpretation of results. Lastly, further research is needed to determine if our findings extend beyond the specific US adult population studied.

## Conclusion

In summary, our study found an association between butyrate consumption and cognitive performance among a nationally representative cohort of older adults in the United States of America. The findings hint at the possibility of a causal relationship, which warrants further investigation through large-scale prospective studies. Our results indicated that elevating butyrate levels, whether through dietary means or otherwise, could potentially contribute to maintaining brain function during aging.

## Data Availability

The raw data supporting the conclusions of this article will be made available by the authors, without undue reservation.
