# Supplementary material for: Higher dietary butyrate intake is associated with better cognitive function in older adults: evidence from a cross-sectional study
Source: Front Aging Neurosci. 2025 Mar 28;17:1522498. doi: 10.3389/fnagi.2025.1522498 (PMC11985818; doi:10.3389/fnagi.2025.1522498)
Supplement: Supplementary file 1 [file Table_1.DOCX]

| Supplementary Table 1. Subgroup analysis of the association between dietary butyrate intake and DSST | | | |
| --- | --- | --- | --- |
|  | β (95% CI) | P | P for interaction |
| Age |  |  | 0.100 |
| <70 | 2.350(1.008, 3.691) | <0.001 |  |
| ≥70 | 1.064(-0.570, 2.698) | 0.202 |  |
| Gender |  |  | 0.526 |
| Male | 1.871(0.329, 3.413) | 0.017 |  |
| Female | 1.606(0.204, 3.009) | 0.025 |  |
| BMI |  |  | 0.018 |
| Obese | 3.377(1.752, 5.002) | <0.001 |  |
| Normal | 0.132(-2.119, 2.383) | 0.908 |  |
| Overweight | 1.253(-0.482, 2.988) | 0.157 |  |
| Underweight | 5.004(-14.463,24.471) | 0.563 |  |
| Race |  |  | 0.650 |
| Non-Hispanic white | 0.548(-0.857, 1.952) | 0.444 |  |
| Non-Hispanic black | 2.348(0.164, 4.533) | 0.035 |  |
| Mexican American | -0.824(-4.375, 2.727) | 0.647 |  |
| Others | 3.355(0.752, 5.958) | 0.012 |  |
| Hypertension |  |  | 0.313 |
| Yes | 2.131(0.773, 3.489) | 0.002 |  |
| No | 1.118(-0.508, 2.744) | 0.178 |  |
| Diabetes |  |  | 0.564 |
| No | 1.791(0.598, 2.985) | 0.003 |  |
| Yes | 1.327(-0.793, 3.446) | 0.219 |  |
| Cardiovascular disease |  |  | 0.981 |
| No | 1.614( 0.457, 2.772) | 0.006 |  |
| Yes | 2.121(-0.213, 4.456) | 0.075 |  |

| Supplementary table 2. Subgroup analysis of the association between dietary butyrate intake and Z score | | | |
| --- | --- | --- | --- |
|  | β (95% CI) | P | P for interaction |
| Age |  |  | 0.004 |
| <70 | 0.137(0.073, 0.202) | <0.001 |  |
| ≥70 | 0.017(-0.068, 0.103) | 0.694 |  |
| Gender |  |  | 0.547 |
| Male | 0.066(-0.010, 0.142) | 0.089 |  |
| Female | 0.111(0.039, 0.182) | 0.002 |  |
| BMI |  |  | 0.016 |
| Obese | 0.154(0.072, 0.236) | <0.001 |  |
| Normal | -0.02(-0.132, 0.092) | 0.727 |  |
| Overweight | 0.090(0.003, 0.176) | 0.042 |  |
| Underweight | 0.245(-0.967,1.458) | 0.647 |  |
| Race |  |  | 0.989 |
| Non-Hispanic white | 0.039(-0.033, 0.110) | 0.290 |  |
| Non-Hispanic black | 0.164(0.048, 0.281) | 0.006 |  |
| Mexican American | -0.006(-0.186, 0.174) | 0.945 |  |
| Others | 0.116(-0.007, 0.239) | 0.064 |  |
| Hypertension |  |  | 0.049 |
| Yes | 0.130(0.062, 0.198) | <0.001 |  |
| No | 0.022(-0.058, 0.103) | 0.587 |  |
| Diabetes |  |  | 0.934 |
| No | 0.082(0.024, 0.141) | 0.006 |  |
| Yes | 0.098(-0.013, 0.210) | 0.083 |  |
| Cardiovascular disease |  |  | 0.392 |
| No | 0.092(0.035, 0.149) | 0.002 |  |
| Yes | 0.041(-0.083, 0.165) | 0.516 |  |
